# Principles for pandemics: COVID-19 and professional ethical guidance in England and Wales

**DOI:** 10.1186/s12910-021-00643-1

**Published:** 2021-06-24

**Authors:** Helen Smith, Peta Coulson-Smith, Mari-Rose Kennedy, Giles Birchley, Jonathan Ives, Richard Huxtable

**Affiliations:** 1 Centre for Ethics in Medicine, Population Health Sciences, Bristol Medical School, University of Bristol, Canynge Hall, 39 Whatley Road, Bristol, BS8 2PS UK; 2 Clinical Ethics and Law at Southampton (CELS), Faculty of Medicine, University of Southampton, MP127, Room 2013, Southampton, UK; 3 Centre for Cancer Immunology, Southampton General Hospital, University of Southampton, Tremona Road, Southampton, SO16 6YD UK

**Keywords:** Clinical ethics, COVID-19, Coronavirus, Ethical guidance, Professional guidance

## Abstract

**Background:**

During the arrival of the COVID-19 pandemic, various professional ethical guidance was issued to (and for) health and social care professionals in England and Wales. Guidance can help to inform and support such professionals and their patients, clients and service users, but a plethora of guidance risked information overload, confusion, and inconsistency.

**Methods:**

During the early months of the pandemic, we undertook a rapid review, asking: what are the principles adopted by professional ethical guidance in England and Wales for dealing with COVID-19? We undertook thematic content analysis of the 29 documents that met our inclusion criteria.

**Results:**

The 29 documents captured 13 overlapping principles: respect, fairness, minimising harm, reciprocity, proportionality, flexibility, working together, inclusiveness, communication, transparency, reasonableness, responsibility, and accountability.

**Conclusions:**

We intend this attempt to collate and outline the prominent principles to be helpful, particularly, for healthcare practice during the COVID-19 pandemic and, hopefully, for future pandemic planning. We also offer some reflections on the guidance and the principles therein. After describing the principles, we reflect on some of the similarities and differences in the guidance, and the challenges associated not only with the specific guidance reviewed, but also with the nature and import of “professional ethical guidance”.

## Background

As the COVID-19 pandemic unfolded, professional guidance emerged, which aimed to inform and support decision-making by health and social care professionals working in healthcare practice. In the UK, beyond the extant flu pandemic ethical framework [1], COVID-specific guidance was issued from late March 2020, by various professional organisations concerned with health and social care.

These efforts to provide guidance were welcome, as guidance should help to ensure that professionals’ decisions are clear, consistent, and fair [2]. Unfortunately, however, the status, import and scope of guidance is not always clear: guidance can take varying forms, have varying force, be issued by a variety of organisations or individuals, and be issued to various audiences. Moreover, the abundance of COVID-specific guidance threatens to overload and confuse professionals, especially if recommendations conflict [2].

Mindful of the latter concern particularly, we undertook a rapid review of the emerging guidance. Our focus was on professional *ethical* guidance directed at professionals providing health care in England and Wales and the ethical principles they advance. Our efforts to identify, collate, and summarise the ethical guidance—and specifically the principles therein—are intended primarily to help healthcare professionals, by informing them of the main recommendations, which should hopefully support them when making ethical judgments in their practice during the COVID-19 pandemic and in the long-awaited recovery from this crisis.

After defining some key terms and outlining our approach, we describe the 13 principles we have identified: respect; fairness; minimising harm; reciprocity; proportionality; flexibility; working together; inclusiveness; communication; transparency; reasonableness; responsibility; and accountability. As we note in the discussion, the guidance usefully converges on (overlapping) principles, although there are points of difference, which threaten to confuse professionals and lead to inconsistent (and even, according to some critics, potentially unlawful) practice.

## Methods

Our central question was: what are the principles adopted by professional ethical guidance in England and Wales for dealing with COVID-19? Our focus was on England and Wales as, although there are some legal differences between the two countries, they form a single jurisdiction. Whilst PEG may not always be legal or regulatory in nature, we presume its substance will (and should) reflect the applicable law in the given jurisdiction, hence our focus on a single legal system. Before outlining our approach to the review, we first define two of the key terms, “professional ethical guidance” (PEG) and “principle”.

For the purposes of this review, we defined PEG as a published document, which:

- is aimed at health and social care professionals or specific groups thereof;
- aspires to guide the practice of those to whom it is directed;
- is prepared, issued, and/or endorsed by a professional organisation; and
- is ethical guidance i.e. articulating the ethical attitudes that should be cultivated and/or ethical behaviours, motivations or values that should be adopted by the professionals to whom it is addressed.

The normative force of such guidance can vary [3]. Some PEG will have regulatory force, such as guidance issued by the General Medical Council (GMC) or Nursing and Midwifery Council (NMC). Other organisations—like those in the Academy of Medical Royal Colleges or trade unions (like the British Medical Association (BMA))—will lack regulatory force, but they are nevertheless influential in healthcare practice [2]. Mindful of the potential for such influence, and thus their impact in practice, we sought to cover all such guidance that we could discover within the time frame.

Our aim was to distil the ethical principles adopted in the PEG we reviewed. As Beauchamp and Childress note, the ethical lexicon “encompasses many standards of conduct, including moral principles, rules, rights, and virtues” [4, p. 3]. Accounts of these terms, and the relationships between them, vary [4, pp. 8–9], [5, 6]. Here, we essentially follow Beauchamp and Childress in seeing ethical principles as “general norms”, which seek to guide action, but which also “leave considerable room for judgment in many cases” [4, p. 13].

Led by our central question, we aimed to identify, collate, and summarise PEG addressed at healthcare during the initial stage of the pandemic, including PEG for social care where this was pertinent to health. Beyond our central question, we asked:

- Which organisations issued PEG?
- For which professionals have PEG been issued?
- When had PEG been issued and, where applicable, revised?
- Which ethical principles (or values or positions) had been advanced in PEG?
- What were the similarities and differences between the PEG offered?

Supported by a time-limited small project grant, we undertook a rapid review: “a type of knowledge synthesis in which components of the systematic review process are simplified or omitted to produce information in a short period of time” [7]. Given the time pressures, this approach has understandably been taken elsewhere during the pandemic [8].

PEG was included if it:

- was prepared, issued, and/or endorsed by a professional organisation(s) concerned with health or healthcare;
- was explicitly (whether wholly or in part) directed to professionals making healthcare decisions;
- was explicitly framed as ethical guidance i.e. articulated the ethical attitudes that should be cultivated and/or ethical behaviours, motivations or values that should be adopted by the professional to whom it is addressed;
- was national, covering the jurisdiction of England and Wales;
- was in English, in the public domain and accessible;
- dealt specifically with COVID-19;
- had been published between 1 January 2020 and 12 May 2020.

PEG was excluded if it did not meet the inclusion criteria.

To obtain PEG, first, Google searches were performed, which were revised and refined using search terms and combinations thereof.^1^ Second, websites of professional organisations known to issue guidance for professionals making healthcare decisions were checked for PEG (Box 1). (As we will discuss later, specifying the “professional organisations” to include is challenging.) Third, ‘snowballing’ for guidance was performed on retrieved PEG’s references to capture any other qualifying PEG.

**Box 1:** Organisations checked for PEG

Each of the Royal Colleges associated with medical practices
Care Quality Commission (CQC)
Department of Health and Social Care (DHSC)
General Medical Council (GMC)
Nursing and Midwifery Council (NMC)
Health and Care Professions Council (HCPC)
NHS England
NHS Providers
Joint NHS England and NHS Improvement coronavirus website
Professional Standards Authority for Health and Social Care
The National Institute for Health and Care Excellence (NICE)
Institute of Medical Ethics
Nuffield Council on Bioethics
Resuscitation Council (UK)
Large employment unions as specific to healthcare:
British Medical Association (BMA)
Royal College of Nursing (RCN)
Unison

The inclusion criteria were then applied to all items returned. Included documents were subjected to conventional thematic analysis [9, 10]: they were initially descriptively coded to capture meaningful content that spoke to our research questions, and the codes were then developed into themes that best captured the substance of the guidance overall. The technique of constant comparison was used to ensure that differences and similarities could be captured.^2^

## Results

### Overview

Twenty-nine PEG documents were included, from which we identified 13 principles, described below. Table 1 lists the included PEG, specifying the issuing organisation(s), title, and date of first publication (where stated).

**Table 1:** PEG in overview

Issuing organisation(s)	PEG title	Original publication date
BMA	COVID-19—ethical issues. A guidance note	1 April 2020
BMA, Care Provider Alliance, Care Quality Commission, Royal College of General Practitioners (RCGP)	Joint statement on advance care planning	30 March 2020
DHSC	COVID-19: ethical framework for adult social care	19 March 2020
DHSC	Guidance: Coronavirus (COVID-19): looking after people who lack mental capacity	9 April 2020
Faculty of Forensic and Legal Medicine	Position statement on the management of suspected COVID-19 cases in police custody	16 March 2020
Faculty of Intensive Care Medicine	Decision making for critical care in the context of COVID-19 background to the NICE guidance	25 March 2020
The Faculty of Intensive Care Medicine, Intensive Care Society, Association of Anaesthetists and Royal College of Anaesthetists	Clinical guide for the management of critical care for adults with COVID-19 during the coronavirus pandemic	8 April 2020
GMC	Ethical guidance: coronavirus: your frequently asked questions	(2020)
GMC	Remote consultations	(2020)
General Chiropractic Council, General Dental Council, GMC, General Optical Council, General Osteopathic Council, General Pharmaceutical Council, Health and Care Professions Council, Nursing and Midwifery Council, Pharmaceutical Society of Northern Ireland, Scottish Social Services Council, Social Work England	Joint statement from chief executives of statutory regulators of health and care professionals: How we will continue to regulate in light of novel coronavirus (COVID-19)	3 March 2020
GMC Joint Statement: Chief Medical Officers for Wales, Scotland, Northern Ireland, and England, National Medical Director NHS England and NHS Improvement, Medical Director and Director of Education and Standards GMC	Joint statement: supporting doctors in the event of a COVID-19 epidemic in the UK	11 March 2020
HCPC	Applying our standards	(2020)
Intensive Care Society (ICS)	Clinical guidance: assessing whether COVID-19 patients will benefit from critical care, and an objective approach to capacity challenges	(2020)
NHS employers	Indemnity and Litigation: the current position around continued indemnity arrangements and assurances during the COVID-19 pandemic	22 April 2020
NICE	COVID-19 rapid guideline: critical care in adults: NICE guideline [NG159]	20 March 2020
NMC and GMC	Statement on advance care planning during the COVID-19 pandemic, including do not attempt cardiopulmonary resuscitation (DNACPR)	15 April 2020
RCGP	Ethical guidance on COVID-19 and primary care	5 May 2020
Royal College of Nursing (RCN)	COVID-19 guidance on DNACPR and verification of death	(2020)
RCN	Personal protective equipment (PPE) and COVID-19	(2020)
RCN	Clinical guidance for managing COVID-19: “Ethical guidance/rationing health and care support” section	(2020)
RCN	Guidance for members: refusal to treat due to lack of adequate PPE during the pandemic	9 April 2020
Royal College of Paediatrics and Child Health (RCPCH)	Ethics framework for use in acute paediatric settings during COVID-19 pandemic	16 April 2020
Royal College of Physicians of London (RCP)	Ethical dimensions of COVID-19 for frontline staff	31 March 2020
Royal College of Psychiatrists	COVID-19: ethical considerations	(2020)
Royal Pharmaceutical Society	RPS guidance on ethical, professional decision making in the COVID-19 pandemic	8 April 2020
UK Clinical Ethics Network (UKCEN)	Principles to consider in decision-making	31 March 2020
Unison	COVID-19 advice for those working in critical care	(2020)
Wales Disability Reference Group	Coronavirus (COVID-19) and the rights of disabled people in Wales	8 April 2020
Welsh Government	Coronavirus: ethical values and principles for healthcare delivery framework	12 April 2020

### The principles

#### Respect

Respect means “holding a view of the person as a whole, taking into account their rights, wishes and feelings as a unique individual” [11]. As first articulated in the Government pandemic flu guidance, the principle of respect requires professionals to “keep people as informed as possible; give people the chance to express their views on matters that affect them; respect people’s personal choices about care and treatment” [1, 12–14].The principle, which broadly echoes the familiar principle of respect for autonomy and thus aligns with the Kantian injunction to treat people as ends-in-themselves [15], connects with other principles, such as inclusiveness and fairness. Regarding the latter, the ICS emphasises that “all patients must be treated with respect and without discrimination, because everyone is of equal value” [16].

#### Fairness

The principle of fairness, which also originates in the pandemic flu guidance [1], is broad [17], reportedly capturing neighbouring terms and concepts like equal respect and distributive justice, although the precise relationships between them vary depending on the PEG in question.^3^ The principle requires that people are treated fairly and equally, that processes are fair, and that decision-making is consistent.

First, “everyone matters equally, so people with an equal chance of benefiting from healthcare resources should have an equal chance of receiving them”; originating in the pandemic flu guidance [1], this requirement recurs in subsequent PEG [11, 12, 14, 16]. However, “this does not mean that everyone will be treated the same” [11, 12, 17, 18]. What it does mean is that individual rights—including legal rights, such as those conferred by the Equality Act 2010 or human rights instruments—should be respected [12, 19, 20]. Furthermore, the principle of fairness requires the avoidance of unjust discrimination: blanket policies are unethical and unlawful [11, 21], and “reasonable adjustments” should be made, for example, to ensure that those with disabilities are “on an equal footing to those who are not disabled” [12, 22].

Some guidance also recognised, however, that egalitarianism may need to give way to alternative accounts of fairness if resources (for example, in intensive care) become scarce. The BMA, in particular, was prepared to adopt a utilitarian approach, where “The focus of health professionals’ attention during triage will be on delivering the greatest medical benefit to the greatest number of people” [12]. For the BMA, this meant that priority should be given to those with “the capacity to benefit quickly” [12], a criterion adopted in some subsequent guidance [18, 23].

Second, fairness is an aspect of “procedural ethics”, which requires decisions to be made “openly, accountably, transparently, by appropriate bodies and with full public participation”, with decision-makers seeking outside opinions and advice when needed [12]. The principle thus overlaps with such other principles as respect, reasonableness, transparency, and inclusiveness [13, 16, 17]. Finally, fairness also requires consistency: decisions (for example, about resource allocation) should be consistent with one another [12], and with legal, ethical or other guidance [14, 17, 22], and consistency may also require decision-making to be collective and/or guided by a decision-making framework [24].

#### Minimising harm

Minimising harm is “defined as striving to reduce the amount of physical, psychological, social and economic harm that the outbreak might cause to individuals and communities” [12]. National, collective efforts—such as hand washing and ‘social distancing’—can help minimise harm [13]. To further limit the spread of infection [12, 13], decision-makers in health and social care should: co-operate, learn from, and share local and national experiences as understanding develops [11, 13]; enable informed decision-making [12]; and minimise the impact of the pandemic on other services essential for the population’s survival and wellbeing [11]. Clinicians are also advised to minimise harm by reducing the risk of complications when someone is unwell [13], through avoiding the inappropriate provision or omission of treatments or interventions [11]: “patients should receive the interventions that are most likely to benefit them” [16]. Should services become overwhelmed due to the pandemic, decision-makers are advised to focus on minimising the loss of life [16]. Clinicians are also advised to minimise harm to themselves, for example, by modifying “usual procedures to accommodate and minimise risk” [25], such as through the use of personal protective equipment (PPE) [12, 14, 17, 22, 26–30].

#### Reciprocity

Reciprocity means that “those who take on increased burdens should be supported in doing so” [12]. First featuring in the pandemic flu guidance [1], reciprocity appears in the later guidance, either as a principle [11, 12, 14], or as a concept supporting a value [17]. Reciprocity requires that “risks and burdens are minimised as far as possible for all, responding proportionately to the risk” [11]. Reciprocity thus connects with the principles of minimising harm and proportionality and, more generally, with the concept of mutual exchange [14], as well as the professional’s duty of care to their patients or service users [17]. Meeting the duty may expose the professional (or their loved ones) to risk, so they are also owed an obligation (for example, by employers) to ensure the risks are mitigated, for example via the provision of PPE and training in its use [12, 14]. The importance of reciprocity was expressed in divergent ways: one rationale for owing reciprocity to healthcare workers is that protecting professionals means protecting patients [14]; another is that reciprocity “shows solidarity while protecting the public from harm” [17].

#### Proportionality

Proportionality is sometimes captured by other principles, like inclusiveness [13], but also features as a distinct principle [18]. Proportionality tends to be cited in relation to the impact of decisions, risk/benefit calculations and communication. The principle requires that different considerations—particularly benefits and risks—be balanced [14]. As such, the goal of protecting the public from harm should be balanced against the impact on individual rights [18], or on particular groups [13]. Risks might not entirely be eliminated, but any risk that is taken should be proportionate to the benefit that might be accrued. Proportionality also influences what and how information is imparted to patients and their carers: information should be accurate [14], but may need to be tailored, so that both the benefits and the risks of communicating the information are considered [18].

#### Flexibility

“Flexibility in a pandemic is key”, according to the RCP [17]. The DHSC describes this principle “as being responsive, able, and willing to adapt when faced with changed or new circumstances” [11–14, 17, 18, 22, 26]. The principle thus requires agility and adaptability when (for example) new information emerges, demands vary, or resources become depleted [13, 18]. Requirements are imposed on professionals, organisations and regulators. Professionals should be prepared to work differently, in new roles or places [17, 18, 22, 26], work collaboratively, including with patients and service users, and adapt plans when necessary [11, 13, 18], which may mean varying how they meet their professional obligations and duty of care [17, 18, 26, 31]. Organisations should also be flexible in their plans, policies and protocols [12, 13, 18]; this may mean redeploying staff, which requires staff to be supported, trained, and, ultimately, kept safe [13, 17, 26, 32]. Regulators, meanwhile, should take due account of the context in which a professional is working during the pandemic [26, 31].

#### Working together

The concept of working together aligns with the DHSC’s principle of community, which involves “a commitment to get through the outbreak together by supporting one another and strengthening our communities to the best of our ability” [13]. Healthcare is a multidisciplinary endeavour, requiring cooperation, collaboration, and support, across disciplines and organisations [11–13]. Everyone will be affected by the pandemic, so everyone ought to work together in planning, responding to, and coping with the effects of the pandemic [11–14, 16, 18]. Sharing information will help to ensure such goals are met, for example, by sharing knowledge about the benefits and risks of a treatment, to help others [11, 13, 14, 18]. More experienced clinical professionals should also “collaboratively make the key decisions, providing direction to more junior staff” [18]. Professionals should also be prepared to involve those whom decisions will affect; for example, persons with disabilities must be involved in the development of guidance and decisions which affect those persons [19].

#### Inclusiveness

Inclusiveness is defined by the DHSC as “ensuring that people are given a fair opportunity to understand situations, be included in decisions that affect them, and offer their views and challenge. In turn, decisions and actions should aim to minimise inequalities as much as possible” [13]. The principle links with fairness, respect and working together [19]. Inclusiveness is considered important in the pandemic, when some people—such as those with disabilities—might be more disadvantaged than others [13, 19]. The principle requires decision-makers to be engaged in an active, two-way, and accessible process, which involves individuals and, where appropriate, their carers, families, and communities [13, 17, 19].

#### Communication

“Decisions must be taken in consultation and discussion with the patient during this pandemic, wherever possible”, says the RCN [20], and multiple sources affirm the importance of good communication, including with colleagues [18, 23, 30, 33–35]. The guidance conveys five positive obligations. First, professionals should be polite, considerate, and sensitive when communicating with patients and their loved ones [23, 33, 34], an obligation that the GMC also imposes on clinical leaders when communicating with colleagues [26]. Second, communication should be clear and accessible, including within teams [26]. Professionals should accordingly seek to make arrangements “to meet service users’ and carers’ language and communication needs” [33]. This requires professionals to be alert to possible barriers to effective communication, such as the stress caused by the pandemic [33], as well as the use of PPE and remote technologies, either of which may be “a disabling barrier” that inhibits (for example, non-verbal) communication [28, 33]. Third, professionals should, as the HCPC states, “give service users and carers the information they want or need, in a way they can understand” [33]. Patients, carers and colleagues should therefore be informed, which may require the professional to ascertain the person’s understanding, or otherwise to support the person to ensure that they can understand [18, 23, 33, 34]. Fourth, professionals should be prepared to listen [33]. Finally, professionals should be prepared to document (especially) serious decisions, such as those concerning the withholding or withdrawing of treatment [20, 30]. Guidance also suggests at least two negative obligations. First, professionals should not avoid difficult conversations, such as those concerning advance care planning, treatment escalation plans, or cardiopulmonary resuscitation, the latter of which is also a legal requirement [30, 34, 35]. Second, despite the anxiety and stress caused by the pandemic, professionals should not “tolerate unacceptable abuse” [33].

#### Transparency

Transparency calls for individual and organisational openness. The principle requires professionals to be open when communicating with patients, service users, families, and colleagues, which includes candour when things go wrong, and being open about the reasons for a decision, as well as who is making the decision [12, 13, 34, 36]. The principle links with various others, including fairness [13, 17], inclusiveness [12, 13, 17], communication [12, 13, 17, 18, 20, 22, 24, 34], and accountability [12, 13, 17, 18]. Transparency has instrumental value, since it helps to ensure that decisions are lawful [20], that public trust is fostered [17, 33], that the public understand and accept decisions 12, 16, 17], and that professionals and organisations can learn from mistakes [33]. Openness is seen as particularly important if there needs to be a shift from resource-unconstrained to resource-constrained decision-making [24], especially if there may be differential treatment [13], and in discussions about cardiopulmonary resuscitation and other care options [34].

#### Reasonableness

Connecting with such principles as fairness, inclusiveness, flexibility, reciprocity, and communication, the principle of reasonableness is defined by the DHSC as “ensuring that decisions are rational, fair, practical, and grounded in appropriate processes, available evidence and a clear justification” [13]. As such, reasonable decisions will be “grounded in reason” [18], practical [12], and have a clear justification that can be explained [13, 26].^4^ They should also follow “a reasoned decision-making process” [18], which accounts for (and allows time to consider) stakeholders’ contributions [13]. The process should ensure that decisions are not only evidence-based [12, 13, 17, 18, 20, 38], but also compliant with law [16], professional standards [31], and relevant guidance [17, 20].

#### Responsibility

Professionals and organisations are required to fulfil their roles responsibly [13, 25, 26]. The relevant responsibilities may be stipulated by law, a professional regulator, or (for some organisations) a particular institution [13, 32]. The ensuing responsibilities may in turn be owed to various stakeholders. The Government is said to owe responsibilities to the population it serves [12, 16], but the focus in the PEG tends to be on individual decision-makers (such as clinicians), who have responsibilities towards their patients, place of practice, families, friends, and/or the public [14, 26], and on organisations, which will additionally owe responsibilities to their staff [12, 13, 31, 37].

As previously noted, there is some recognition that responsibilities may vary or even be lifted during the pandemic [13], but PEG consistently conveys that established responsibilities will typically remain, for example, regarding child safeguarding [26], use of social media [33], respecting confidentiality [22, 26, 39], and acting in the best interests of incapacitated patients [39, 40]. Moreover, PEG affirms that clinicians continue to owe a duty of care to their patients, despite the pandemic, or the setting in which the patient is being cared for [17, 22, 25, 26]. Linking with the principles of flexibility and reciprocity, that duty may require a clinician to be re-deployed [22], but, whether or not this is the case, employers will continue to owe a duty to professionals to protect them, for example through the provision of adequate PPE [17] (which is linked to reciprocity).

There are, as such, responsibilities owed by professionals and organisations alike. Professionals should ensure they work within their “scope of practice”[32]—i.e. within the limits of their knowledge, skills and experience [38]—but should also recognise that this responsibility may sometimes be outweighed by patients’ needs [18]. Professionals should also be prepared to receive and—along with organisations—provide training, guidance, induction and supervision [12, 18, 20, 26, 32]. Equally, professionals should be willing to receive support and, along with organisations, provide it, since it “will help reduce staff exhaustion and moral injury” [26, 41]. This responsibility to receive and provide support also arises from the principles of proportionality and reciprocity [12–14, 18]. Forms of support include resources, maintaining appropriate working environments [13, 20, 26], psychological support [12], and processes for handling ethical challenges [13], (such as access to Clinical Ethics Committees [12, 16]). Some guidance emphasises the importance of providing support for difficult decisions in particular, such as decisions about withholding or withdrawing life-sustaining treatment or prioritising access, which should not be left to individuals alone [16, 17].

#### Accountability

The DHSC defines accountability as “holding people, and ourselves, to account for how and which decisions are made. In turn, this requires being transparent about why decisions are made and who is responsible for making and communicating them” [13]. Accountability overlaps not only with responsibility, transparency and communication, but also with respect, since accounting for one’s actions may signal respect for the affected person(s), and with fairness, insofar as accountability is an aspect of a “fair process” [12]. The principle also has instrumental value, as it fosters public trust in, and acceptance of the decisions made by, the professions and professionals [12, 18, 26]. Professionals may be held to account by reference to their adherence to their profession’s standards [13, 17, 26]. Professionals should be able to explain and justify their decisions [13, 17, 25, 26, 29, 42, 43], which may be aided by keeping clear and contemporaneous records [13, 18, 26, 29, 42, 43]. If professionals are later called to account, for example because a concern is reported, organisations and regulators should in turn “take into account factors relevant to the environment in which the professional is working, including relevant information about resources, guidelines or protocols in place at the time” [31].

## Discussion

Having identified pertinent PEG and summarised the principles therein, here we further reflect on what “professional ethical guidance” is and how it may help or hinder professionals (and, indeed, their patients and service users). These reflections focus on the key messages that emerged for us, whilst also acknowledging some of the limitations of not only our attempt to explore professional ethical guidance, but also arguably any attempt to do so.

### What is “professional” ethical guidance?

Clinical guidance emerged rapidly as the COVID-19 pandemic appeared in the UK. Whilst such guidance might implicitly adopt ethical positions, explicitly ethical guidance also appeared, amidst mounting calls for ethical leadership from the respective UK governments [44]. The Scottish Government issued an ethical framework for health and social care on 3 April 2020 [45], which was followed on 12 April by an ethical framework for healthcare issued by the Welsh Government [11].

Ethical guidance for healthcare in Northern Ireland came later, in a document issued by the Department of Health on 21 September 2020 [46].

Turning to England, the DHSC had issued an ethical framework for adult social care on 19 March [13], although authoritative ethical guidance for healthcare seemed conspicuous by its absence. There was, however, an ethical framework for dealing with a flu pandemic already in place in England, which had first been issued in 2013 and revised in 2017 [1]. The Moral and Ethical Advisory Group (MEAG) had also been formed, to provide “independent advice to the UK government on moral, ethical and faith considerations on health and social care related issues” [37]. Its work began in earnest as the COVID-19 pandemic took hold and its terms of reference, composition, and the minutes of its meetings are (now) publicly available [47]. At its eighth meeting on 22 April 2020, MEAG hoped to be commissioned by the Chief Medical Officer (CMO) of England to draft “a statement of ethical principles … to be consistent with the national ethical framework” [47]. However, by the ninth meeting on 29 April, the CMO had “advised against MEAG working to produce a document of principles beyond what was already in place in the ethical framework for pandemic planning as this might crowd out the capacity of MEAG to consider detailed issues on which advice was being sought” [47].

The authoritative professional ethical guidance was therefore piecemeal and, particularly in England, did not appear to cover every healthcare professional. Perceived gaps in the authoritative ethical guidance, which was available at the time, were nevertheless filled by numerous national, regional, and local organisations, whose authority and influence varied. Here, however, a general question arises about what counts as a “professional organisation” and the status or import of any “professional ethical guidance” it issues—and thus about which organisations and which guidance our review (or similar reviews) should seek to include.

We focused on guidance for professionals who make healthcare decisions that was issued by professional organisations pertinent to these individuals and groups. We sought to keep a sharp focus on healthcare but soon appreciated that some organisations that are focused on healthcare also include social care—and therefore such professionals—in their purview. Retaining a primary focus on healthcare, we nevertheless included guidance such as that issued by the DHSC, albeit (in that instance) directed specifically at social care professionals.

Including the DHSC seemed merited, not least because its guidance will be *authoritative*, and thus binding on professionals. There are, of course, many such authoritative sources: guidance from the law (for example, in a Code of Practice accompanying an Act of Parliament), the government (such as a statement from a Ministerial Department like the DHSC), and the nine statutory regulatory bodies overseen by the Council for Healthcare Regulatory Excellence (which include the GMC and NMC) will occupy this category [2]. Sources that do not occupy this category may nevertheless be *influential* and might also be worth heeding (and thus including). Organisations like the BMA have influence not only over their members, but, sometimes, over the position adopted in authoritative guidance [2].

Other organisations complicate the picture further, as it is arguable they do not comfortably qualify as “professional organisations” as such. Some, such as UKCEN, will have healthcare (and such professionals) as their central concern and, despite lacking any official status, will be the key organisation offering support and sometimes guidance in their particular sector (in this case, to members of clinical ethics support services, such as Clinical Ethics Committees). Others, such as the Nuffield Council on Bioethics, offer guidance to healthcare professionals and have undoubted influence in practice, but again would not necessarily be considered “professional organisations”.

As such, we are alert to the challenges associated with identifying a qualifying “professional organisation” in a review of this nature. This is not merely a methodological challenge: a landscape populated by numerous such groups, issuing guidance of varying force or import, can present difficulties for professionals practising under pressure who wish to know whose steer to follow. The explosion in guidance aimed at COVID-19 is potentially a positive development, but this nevertheless threatened to defeat its primary goal—to *guide*.

### Does the professional ethical guidance help to guide professionals?

As the guidance multiplied, concerns were voiced about the confusion that might ensue for professionals and their patients, clients, and service users. If it is to achieve its primary goal, guidance should indeed guide, which at least requires it to be accessible, clear and consistent [6, 48, 49].^5^ In this regard, there is some welcome news, but also some cause for concern.

The welcome news is that the emerging picture is more coherent and convergent than had been feared. First, the PEG advocate many common principles: sometimes the language is identical, but even when it differs, the same or similar concepts appear to be in view. There is also encouraging evidence of joint-working or at least cross-referencing between different PEG. For example, the pandemic flu guidance informed various COVID-19-specific guidance, not only explicitly [12–14, 18], but also implicitly [11]. COVID-specific PEG also informed later such guidance: the first of this guidance, issued by the RCP [17], informed not only later ethical guidance [16, 23, 26], but also more overtly clinical guidance, such as NICE’s position on critical care for adults [52]. Similarly, the BMA guidance was also drawn on [12], not only in the latter document, but also in various ethical guidance [14, 16, 18, 23, 24, 26]. In the opposite direction, some (overtly) clinical guidance—in particular, the NICE guidance [52]—also featured in various PEG [16, 24, 26].

Second, the principles are ‘imbricated’, and thus (perhaps helpfully) overlap in various ways. For example, according to the guidance, reciprocity connects with proportionality, which overlaps with minimising harm and inclusiveness; inclusiveness overlaps with working together, respect, and fairness; fairness, along with (amongst others) inclusiveness and communication, then overlaps with transparency, reasonableness, and accountability; and accountability links with responsibility, proportionality, and reciprocity. Notwithstanding the fact that the precise nature of the purported relationship between principles is not always made clear, this imbrication and cross-referencing is positive because it suggests a good deal of consistency, at least at a macro level, across PEG.

However, there are at least three areas of concern. First, the principles are rather abstract, meaning they need further specification—or individual judgment—to apply them in practice. Clinicians are familiar with critically appraising information and making judgments in nuanced, contextualised situations. A framework may help by providing some high-level indications of what is required but, as the MEAG appeared to recognise, more detailed principles and recommendations may also be needed to guide the professional. Some of the guidance certainly sought to provide more detailed specifications of (at least some of) the principles and how these might apply in particular scenarios. This was, for example, evident in the efforts to indicate how the principle of fairness might apply to the provision of intensive care under resource-constrained conditions (e.g. [12]). Furthermore, some of the guidance suggested that (for example) Clinical Ethics Committees could assist in providing not only consistency, but also further specification of the principles [12, 17, 22]. However, as is implicit in the suggestion that professionals have recourse to Clinical Ethics Committees, the guidance documents themselves did not always spell out the principles or how they might apply. This suggests that the outworking of the principles requires judgment—whether by such a Committee or the professional. The interplay between guidance and judgment is complex, and somewhat symbiotic [2], but it seems apparent that the more abstract the guidance, the more professional discretion is required—and with this comes the risk of increasing inconsistency at a micro level, thus undermining the goal of guiding action.

Second, and related to this point, the PEG are not entirely uniform, as they sometimes specify the principles in different ways. For example, there was some variation in how the principle of fairness would apply to a situation in which resources—in particular, in intensive care—were constrained. The BMA was prepared to adopt a utilitarian approach, “delivering the greatest medical benefit to the greatest number of people”, which meant that priority should be given to those with “the capacity to benefit quickly” [12]. Although some organisations adopted this criterion [18, 23], the UKCEN excluded the word “quickly”, instead referring to “the criterion of ‘ability to benefit from therapy’” [24]. As noted above, the authority and influence of these organisations varies, but—particularly for the professional under pressure—such differences have the potential to confuse. The difficulty is probably not knowing that “fairness” is important (most healthcare professionals would not need additional guidance to tell them this), but knowing which decisions, and which trade-offs against other principles, are fair in this situation. An apparently small difference in wording can substantially alter how the principle would apply. Given that it is unclear which account of applied fairness is correct, further guidance might be needed to help navigate the existing guidance. In short, the goal of action-guidance is jeopardised if a professional is offered divergent accounts of how a principle should be applied.

Third, that goal is potentially also threatened by the plurality and imbrication of the principles. Our list is admittedly longer than some of the individual PEG, which opted to cluster some principles: for example, some PEG followed the pandemic flu guidance [1], in including at least six of our principles—respect, inclusiveness, communication, transparency, reasonableness, and accountability—under the single principle of “good decision-making” [12, 14, 17]. The MEAG seemed to favour brevity, noting of its (subsequently abandoned) draft “that some principles are overlapping and could be combined” [47].

It may indeed be possible to re-organise, combine and/or reduce our list of 13 principles. One option would involve separating the principles into primary *substantive* ethical principles and secondary *operational* (or procedural) principles. The primary, substantive principles would most obviously include respect, minimising harm, and fairness—and “fairness” could additionally accommodate such other principles as reciprocity, inclusiveness, transparency, reasonableness, and proportionality. The secondary, operational principles could then include flexibility, working together, communication, responsibility and accountability. However, vexed questions are likely to arise about what counts as a substantive or an operational principle: for example, the pandemic flu guidance seems to view an arguably substantive principle like “respect” as more operational in nature (as an aspect of “good decision-making”). Another question, which we pick up below, concerns whether these are (in some sense) the right principles and how, if at all, they relate to more familiar existing ethical principles, such as respect for autonomy and beneficence. In short, difficulties arise when trying to tease out the nature and substance of the principles and the precise relationships between them, not least given their explicit overlaps.

Neither plurality nor overlap are necessarily problematic—*if* the principles are sufficiently univocal and do not pull the decision-maker in different directions. However, not all the apparently overlapping principles will point in the same direction. For example, in a pandemic, the individualistic principle of respect (distinctive of clinical ethics) lies in tension with the more population-oriented principle of fairness (distinctive of public health ethics). Some guidance has been criticised (and legally challenged) for over-emphasising the latter concern at the expense of the former [48]. But the PEG surveyed generally fails to explicate how competing or conflicting principles (whether these or others) are to be balanced. “Principles can conflict and leave it unclear what one should do in any particular situation”, says Archard, who suggests a need for some sort of ranking of principles [50]. Beauchamp and Childress, the architects of the prominent four principles of biomedical ethics, provide a method—“reflective equilibrium”—for specifying and balancing their principles, which is meant to help a clinician determine how to proceed. This method involves reaching a balance between one’s intuitions, empirical facts and background theory by discarding elements that are not coherent with one another [4]. It may have conceptual and practical shortcomings [51], but at least a method is offered. Some of the PEG may offer some (“meta” or operational) principles for decision-making [12], but none of them appear to provide a clear method for resolving conflicts between principles.

### Is the professional ethical guidance itself ethical?

Inconsistency not only undermines the goal of guidance, but also—for PEG—interferes with its proclaimed *ethical* orientation. Aristotle, for example, essentially saw inconsistency as (formally) unjust, insofar as like cases would not be treated alike [53]. Pluralism was arguably not a concern for Aristotle’s original audience but, in England and Wales today, the “ethical” may be judged in various ways, and determining what counts as *professional ethical* guidance and whether it is indeed “ethical” is challenging.

In advocating what should (or even must) happen, guidance per se is likely to have to adopt a particular ethical position. Even purportedly *clinical* guidance is likely to do so, more or less openly; NICE, for example, reveals something of its ethical orientations by citing the BMA’s ethical position on decision-making when resources are scarce [52]. Yet, whether guidance has adopted the “right” ethical position(s), as judged by some or other ethical standard, is an open question. The NICE guidance was challenged on just such a basis [54]. Moreover, similar challenges have been levelled at (avowedly) professional *ethical* guidance, including that issued by the BMA [55]. The challenge might originate in a different ethical perspective from the one adopted in the guidance; alternatively, guidance might be judged deficient on its own terms. For example, although there are examples of good practice regionally [56], the extent of public involvement in the development of the national PEG is not entirely clear—despite the PEG emphasising such principles as respect, inclusiveness, communication, and transparency. As such, what (dis)qualifies guidance as substantively “ethical” is a source of difficulty.^6^

PEG nevertheless appears to be a distinct source of “ethics”, occupying a complex web comprising not only clinical guidance, but also the perspectives offered by (for example) those working in the academy, stakeholders, and the broader public—and, ultimately, the law. As we noted above, law is an important—and on some accounts the *primary*—source of authoritative guidance [3]. However, the precise relationship between PEG and the law has vexed some lawyers during COVID-19. Early on, Thomas et al. called for national consensus, suggesting that doctors deserve the reassurance of knowing that what they are advising and doing is lawful [57]. Regarding scarcity and triage, Liddell et al. feared that “many legally enforceable rights have been overlooked in the ethically focussed guidelines that have been produced thus far” [58, 59]. One particular area of legal concern is the treatment of patients who lack (mental) capacity; as Parsons and Johal put it, “the pressure to develop rapid national guidance has resulted in [legal] considerations of its application being overlooked, particularly in relation to vulnerable populations” [60]. Although some of the maligned guidance was prepared with legal input, there have accordingly been reports of legal challenges being mounted to some of the (at least, clinical) guidance [2]. If law and guidance are out-of-step, this may invite revisions in one or other domain; pending this, however, professionals might question whether there is a “legal risk” to heeding guidance issued by less authoritative sources [56], thus further frustrating the goal of guiding professional behaviour.

## Conclusion

In addition to the voluminous clinical guidance that has been issued during COVID-19, there has been a proliferation of professional ethical guidance in England and Wales. These are welcome efforts to inform and support health and social care professionals and those whom their practices serve. Many common messages helpfully emerge from the PEG, which broadly converge into 13 overlapping principles: respect, fairness, minimising harm, reciprocity, proportionality, flexibility, working together, inclusiveness, communication, transparency, reasonableness, responsibility, and accountability.

However, the guidance landscape is crowded, confusing and not entirely univocal, prompting concerns about clarity and consistency. PEG might, at worst, not be seen or adopted by professionals, be insufficiently action-guiding due to a lack of specificity, fail to guide by issuing contradictory injunctions, or even provide a questionable steer, whether legally or (perhaps paradoxically) ethically. Questions arise about whether such guidance is indeed “ethical” and about what counts as “professional ethical guidance” and which such guidance professionals should heed. [2] Hopefully, future research will further illuminate the nature of PEG, and the relationships between different types of PEG.

Of course, work is also needed beyond the academy. First, there may still be a case for more authoritative and co-ordinated ethical leadership, which can help to enhance the accessibility, clarity, consistency, and applicability of the principles. A clear national ethical approach will be needed as we move into the recovery phase of the pandemic, which will differ from the initial phase when attention was understandably focused on ensuring that the health service was not overwhelmed. An ethical “roadmap” should guide the restoration of services that have been suspended, ensuring that those without (as well as those with) the virus are appropriately cared for, and the development of treatments for the virus, alongside vaccination efforts [44].

A single national ethical approach stipulating overarching principles might initially be applied to the health services’ recovery work. Rather than each organisation—or local healthcare provider—replicating work to create different (and potentially conflicting) PEGs, a single approach would provide focus and promote a more unified response from clinical stakeholder groups when planning and executing clinical care. Given the convergence of the principles we have identified, some groundwork has been laid for a uniform approach going forward. However, there will be a balance to be struck between clearly articulating a uniform set of principles and allowing for legitimate variation. One major challenge we have identified is the need for further specification of the current principles. Some such specification could usefully be provided at a national level. At the same time, however, room will be needed for legitimate localised, even individualised, specification and judgment.

Second, as we noted above, future PEG should ensure (and demonstrate that) it is informed by the input of stakeholders, including not only health and social care professionals, but also patients, service users and the public at large. This will improve transparency and ensure that it practices the inclusivity that it preaches. We further suggest that this would help to make the guidance substantively more ethical since, in our view, to be ethical, guidance must “appeal to … norms that merit recognition among those to whom they apply”.([61], p. 261).

Finally, despite successful early efforts to prevent the health service from being overwhelmed, resources remain constrained, putting professionals and those they serve under pressure. Further resources are likely to be needed if ethical principles are to be fully realised in practice—or expectations must be carefully managed, so that professionals making healthcare decisions are not set up to fail by the system.

Although it cannot specify all the answers, a uniform and better specified ethical “roadmap” would help to plot the way forward, providing professionals, patients, and the wider public with the reassurance that those making difficult decisions in health and social care have access to clear, consistent, and sound guidance.

## Data Availability

Not applicable.

## Abbreviations

BMA: British Medical Association
CMO: Chief Medical Officer
CQC: Care Quality Commission
DHSC: Department of Health and Social Care
GMC: General Medical Council
HCPC: Health and Care Professions Council
ICS: Intensive Care Society
MEAG: Moral and Ethical Advisory Group
NHS: National Health Service
NICE: The National Institute for Health and Care Excellence
NMC: Nursing and Midwifery Council
PEG: Professional Ethical Guidance
PPE: Personal Protective Equipment
RCGP: Royal College of General Practitioners
RCN: Royal College of Nursing
RCP: Royal College of Physicians of London
RCPCH: Royal College of Paediatrics and Child Health
UK: United Kingdom
UKCEN: UK Clinical Ethics Network

## Footnotes

1 The search terms used (in alphabetic order) were: Advice; Bioethic*; Car*; Care Home; Community; Coronavirus; COVID; COVID-19; Distanc*; Doctor*; Equip*; Ethic*; Guid*; Health; Hospital; Isolat*; Limit*; Medic*; Model*; Nurs*; Pandemic; Policy; Protec*; Scarc*; Social; Treat*. These were combined in different ways, leading to 19 different search strings.

2 As such, we took a qualitative approach to analysis and, for this reason, we do not provide quantitative, numerical data in our findings (such as the incidence of particular principles within and across the guidance).

3 For example, the Welsh Government sees this principle as flowing from the core value “equal concern and respect” [11], while the pandemic flu framework posits “equal respect” as a principle on the same level as the principle of fairness [1].

4 With a legalistic orientation, NHS Employers distinguishes reasonable decisions from reckless ones [37].

5 Some theorists also point to the importance of guidance being capable of performance i.e. not requiring the impossible [49, 50]. Although we will not explore this in depth, concerns may arise here about the PEG. A pandemic might well make it difficult—perhaps even impossible—to honour principles like respect, communication, transparency and inclusiveness, since these potentially require labour- and time-intensive efforts to access and heed the views of patients, service users, and the wider public. Equally, a principle like reciprocity partly requires the provision of adequate PPE to protect professionals—but what if, as sadly transpired, provision is limited or even absent, whether locally or nationally? Resources (whether PPE or other) might also vary between regions or settings, which suggests that some principles may well be operationalizable in one place but not in another.

6 One source of difficulty may be the apparent conflation of the terms “ethical” and “moral”. While this is usually unproblematic in clinical ethics, given its intense focus on the interpersonal aspects of clinical decision-making, it might become problematic when the focus is on collective behaviour. The types of norms we negotiate in a pluralistic society are likely to be different when they apply (as “ethics”) to institutions and societies as opposed to when they apply (as “morals”) to personal conduct.
